# Atlantinone A, a Meroterpenoid Produced by *Penicillium ribeum* and Several Cheese Associated *Penicillium* Species

**DOI:** 10.3390/metabo2010214

**Published:** 2012-02-23

**Authors:** Petur W. Dalsgaard, Bent O. Petersen, Jens Ø. Duus, Christian Zidorn, Jens C. Frisvad, Carsten Christophersen, Thomas O. Larsen

**Affiliations:** 1 Department of Forensic Medicine, University of Copenhagen, Frederik V’s Vej 11, DK-2100 Copenhagen, Denmark; 2 Carlsberg Laboratory, Gamle Carlsbergvej 10, DK-2500 Valby, Denmark; 3 Munkehøjvænge 31, 3520 Farum, Denmark; 4 Institut für Pharmazie, Leopold-Franzens-Universität, Innrain 52, A-6020 Innsbruck, Austria; 5 Center for Microbial Biotechnology, DTU Systems Biology, Building 221, Technical University of Denmark, DK-2800 Kgs. Lyngby, Denmark; 6 Department of Chemistry, University of Copenhagen, Universitetsparken 5, DK-2100 Copenhagen, Denmark

**Keywords:** *Penicillium ribeum*, atlantinone A, andrastin, psychrotolerant, cheese associated fungi

## Abstract

Atlantinone A has been isolated from the psychrotolerant fungus *Penicillium ribeum*. The exact structure of the compound was confirmed by mass spectrometric and 1- and 2D NMR experiments. Atlantinone A was originally only produced upon chemical epigenetic manipulation of *P. hirayamae*, however in this study the compound was found to be produced at standard growth conditions by the following species; *P. solitum*, *P. discolor*, *P. commune*, *P. caseifulvum*, *P. palitans*, *P. novae-zeelandiae* and *P. monticola.* A biosynthetic pathway to atlantinone A starting from andrastin A is proposed.

## 1. Introduction

**Figure 1:**
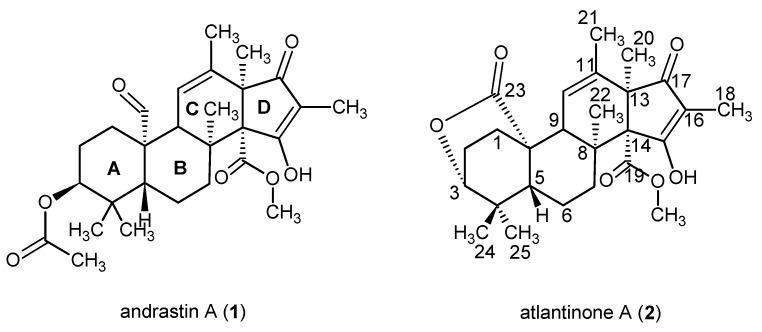
Structures of andrastin A (**1**) and atlantinone A (**2**). Rings A–D are indicated for (**1**) and numbering of the carbon skeleton in (**2**).

Meroterpenoids such as the andrastins and the citreohybridones are interesting as examples of mixed polyketide-terpenoid biosynthesis [1,2,3] and they often have biological activities [3,4,5,6,7]. The andrastins and citreohybridones were originally isolated from hybrid strains of *Penicillium* sp. FO-3929 [1,3] and *P. citreo-viride* B [4]. In the course of our analytical HPLC screening of *Penicillium* species, the characteristic UV-spectrum of andrastin A (**1**) (Figure 1) was detected in many known *Penicillium* species such as the blue cheese mold *P. roqueforti* [8,9], the onion associated mold *P. albocoremium* [10], and psychrotolerant strains of *P. crustosum* from arctic ice [11]. The newly arctic described fungus *P. ribeum* [12,13], however, was found to produce a metabolite with the UV-spectrum of andrastin A, but with a different retention time and molecular mass. The data for the compound matched those of the newly reported compound atlantinone A [14], which we have confirmed by isolation and structure elucidation. HPLC-UV-MS analysis demonstrated that several species within genus *Penicillium* produce the compound under standard laboratory conditions.

## 2. Results and Discussion

An EtOAc extract of *Penicillium ribeum* (IBT 16537) was separated by high-speed countercurrent chromatography (HSCCC), and through UV-guided fractionation the fifth HSCCC fraction was further purified by HPLC to afford pure **2** (6.8 mg).

**Figure 2:**
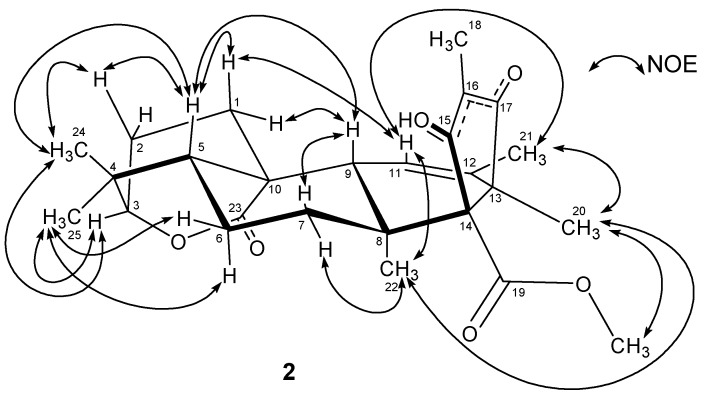
NOE connectivities in (**2**).

Atlantinone A (**2**) exhibited a molecular ion in HREIMS corresponding to the molecular formula C_26_H_34_O_6_(10 degrees of unsaturation). The 2-dimensional structure of **2** was established by interpretation of 1- and 2-dimensional NMR data (see Supplementary data). The relative configuration of **2** was established by NOE experiments (Figure 2) indicating the presence of an *ent*-5α,14β-androstane skeleton with a 10,3α lactone bridge in agreement with the X-ray structure published by Cichewicz’s group [14]. The unusual configuration of **2** is undoubtedly caused by the lactone bridge forcing the oxygen to occupy an α-position.

**Figure 3:**
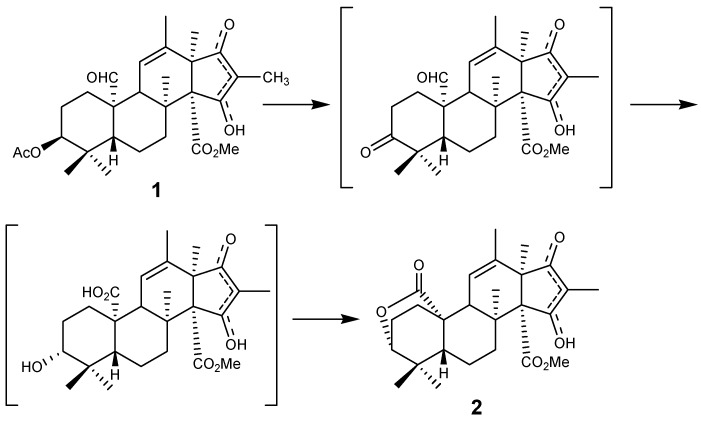
Proposed biosynthetis of atlantinone A (**2**) starting from andrastin A (**1**).

We propose that atlantinone A (**2**) is derived from andrastin A (**1**), which on enzymatic oxidation at C-3 and C-10 could cause the oxygen at C-3 to inverse from β- to α (Figure 3). This reduction may be either stereospecific or non-stereospecific. In the first case the lactone formation would arrest the configuration in the α configuration. In the second type of reduction the α configuration would be effectively removed by lactone formation and would on repetitive oxidation reduction cycles end up completely in the α state. Atlantinone A (**2**) is to our knowledge the first meroterpene with an α configuration in position 3. The citreohybridones [4] and andrastins A-C [1,3] are all reported with β configuration in position 3.

In our previous studies of cheese associated fungi [15] we had observed an unknown peak in several fungal extracts with a UV spectrum very similar to that of atlantinone A. Recultivation on standard cultivation media, followed by extraction and analysis by LC-DAD-MS, indeed confirmed the following species to be atlantinone A producers; *P. solitum*, *P. discolor*, *P. commune*, *P. caseifulvum*, *P. palitans*, *P. novae-zeelandiae* and *P. monticola*, as evident by detection of [M+H]^+^ and [2M+H]^+^ in ESMS in the positive mode similar to those of the authentic standard. We also screened several isolates of *P. hirayamae* for atlantinone A, but were not able to detect it in any of the isolates NRRL 3588, FRR 143, FRR 3330, FRR 1835, or IBT 30340 neither on CYA nor on YES agar.

Since the former five species are often found as contaminants of various types of cheeses [15], one might speculate that atlantinone A can also be detected in cheese products. The compound did not show any inhibitory activities against a panel of bacteria and fungi [16], however it’s structural similarity to the andrastins, previously shown to have potent anticancer drug properties as farnesyltransferase inhibitors [1], indicates that further bioactivity testing is needed in order to establish the potential risk, or possible health beneficial effects of atlantinone A.

While *P. solitum*, *P. discolor*, *P. commune* and *P. caseifulvum* (all members of the section *Fasciculata* [17]) readily produce atlantinone A , *P. hirayamae* (previously incorrectly called *P. citreonigrum*, see [17,18]) from the unrelated section *Sclerotiora* will apparently only produce atlantinone A after treatment with epigenetic modifiers. This difference in ability to express certain secondary metabolites is parallel to our recent findings of orsellinic acid and related compounds being produced at standard conditions by *Aspergillus nidulans* [19], while Schroeckh *et al*. [16], only saw production of these compounds when the fungus was grown in a bacterial co-culture, but in the latter case the same fungus was examined in different laboratories under different conditions.

## 3. Experimental

### 3.1. Fungal Material and Fermentation

*Penicillium ribeum* (IBT 16537) was collected from a soil under a *Ribes* sp. at summit of Eagle Rock, Medicine Bow National Forest in Wyoming by Jens C. Frisvad, 11 September 1994. The fungus was cultivated as five-point mass inoculation on 250 Petri dishes containing CYA at 25 °C for 10 days in the dark. A voucher specimen is located in the collection at the Danish Technical University as IBT 16537. The following fungi were also obtained from the IBT collection; *P. solitum* (IBT 21545, 24251), *P. discolor* (IBT 14472, 22074), *P. commune* (IBT 10763, 26404), *P. caseifulvum* (IBT 15151, 18282), *P. palitans* (IBT 26396, 26410), *P. novae-zeelandiae* (IBT 21932, 22547) and *P. monticola* (IBT 22356, 22478)*.*

### 3.2. Extraction and Separation

The contents of 250 Petri dishes were transferred to four large glass flasks and were extracted at still conditions for 16 h at room temperature with 2.5 L of EtOAc to give 2.4 g of dried extract after evaporation of EtOAc.

The crude extract was directly separated using a PharmaTech CCC-1000 HSCCC [n-Heptan-EtOAc/MeOH/Water (1:1:1:1), mobile phase: upper phase, (T) ° H, 325 mL coils, flow 3 mL/min] connected to Water pumps and diode array detector. Crude fractions (200–250 mg) were dissolved in 30 mL mobile phase (*n*-Heptan/EtOAc (1:1)). The HSCCC separation was repeated several times. Five fractions were collected using UV-guided fractionation. The second, third and fourth fractions were further purified by HPLC on a preparative (7.8 × 300 mm) Waters Symmetry-C18 column (flow rate 4 mL/min) eluted with MeCN/H_2_O (60:40) mixtures to afford pure **2** (6.8 mg).

### 3.3. Apparatus

NMR spectra were recorded in DMSO-*d*_6_on a Bruker 500 NMR spectrometer operated at 500 and 125 MHz for ^1^H- and ^13^C-NMR spectra, respectively and referenced to solvent residual signals and solvent signals at 2.50 ppm (^1^H NMR) and 39.50 ppm (^13^C-NMR). The HPLC data were obtained on an Agilent 1100 HPLC-system using Chemstation software and a Hewlett Packard Hypersil BDS-C18, 4 μm, 4.0 × 100 mm column; flow 1 mL/min. The UV spectra were recorded on a Hewlett-Packard 8452A diode array spectrophotometer. HREIMS mass spectra were recorded on a JEOL JMS_MX/HX 110A. LCMS analysis of plugs was carried out on an Agilent HP 1100 Liquid Chromatograph with a DAD system (Hewlett-Packard) coupled to a LCT TOF mass spectrometer (Micromass, Manchester) with a Z-spray ESI source and a Lock Spray probe. Chromatography was performed on an Phenomenex Luna II C18 column (50 × 2 mm, 3 µm).

### 3.4. Atlantinone A (2)

White powder. [α]
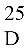
−83.3° (*c* 0.42, MeOH). UV λ_max_ (MeOH) nm (log ε): 260 (sh), 281 (4.56). ^1^H and ^13^C NMR see Table 1 (Supplementary Material). HREIMS *m/z* 442.2254 (calcd for C_26_H_34_O_6_: 442.2355).

## 4. Conclusions

Altogether we have demonstrated that what appears to be a product of a silent pathway in one species, can be a major compound in other species when grown on standard laboratory conditions. Thus, we have shown that atlantinone A previously only expressed by the use of epigenetic modifiers in *P. hirayamae* is readily produced by *P. solitum*, *P. discolor*, *P. commune*, *P. caseifulvum*, *P. palitans*, *P. novaezelandiae* and *P. monticola* on the common media CYA and YES.

## Supplementary Files

Supplementary File 1 PDF-Document (PDF, 366 KB)
