# Supplementary material for: Atlantinone A, a Meroterpenoid Produced by Penicillium ribeum and Several Cheese Associated Penicillium Species
Source: Metabolites. 2012 Feb 23;2(1):214–20. doi: 10.3390/metabo2010214 (PMC3901203; doi:10.3390/metabo2010214)
Supplement: Supplementary File 1 — PDF-Document (PDF, 366 KB) [file metabolites-02-00214-s001.pdf]

Supplementary Material

Atlantinone A, a Meroterpenoid Produced by *Penicillium ribeum* and Several Cheese Associated *Penicillium* Species

Petur W. Dalsgaard [1], Bent O. Petersen [2], Jens Ø. Duus [3], Christian Zidorn [4], Jens C. Frisvad [5], Carsten Christophersen [6] and Thomas O. Larsen [5,*]

Table 1. NMR data of atlantinone A [a] (**2**).

Position	^{1}H	^{13}C
1α	1.33 1H, m	31.5
2α	1.73 1H, m	21.2
3	4.07 1H, s	83.0
4		36.4
5	1.32 1H, m	50.7
6α	1.16 1H, m	21.2
7α	1.93 1H, m	30.9
8		40.7
9	1.80 1H, s	45.4
10		44.8
11	5.35 1H, s	124.4
12		131.8 br.s
13		55.3
14		67.5 br.s
15 [b]		
16		112.0 br.s
17 [b]		
18	1.47 3H, s	6.9
19		170.6
20	1.09 3H, s	15.8
21	1.71 3H, s	19.5
22	1.25 3H, s	16.5
23		174.4
24	1.05 3H, s	27.2
25	0.92 3H, s	21.8
19-OMe	3.48 3H, s	51.1

[a] Measured in DMSO-d_6 (^{1}H at 500 MHz, ^{13}C at 125 MHz) and referenced to solvent residual signals and solvent signals at 2.50 ppm (^{1}H-NMR) and 39.50 ppm (^{13}C-NMR). Numbering is arbitrary and follows the system used for citreohybridone derivatives (*4*); [b] Signals not observed.

Structural elucidation. The ^{1}H-NMR spectrum (Table 1) of **2** showed the presence of an olefinic proton (δ_H 5.35, C_{11}-H), one methoxy group (δ_H 3.48, C_{19}-OMe), one methyl group attached to a double bond (δ_H 1.71, H_3-21), and four methyl groups connected to quaternary carbons [δ_H 0.92 (H_3-25), 1.05 (H_3-24), δ_H 1.09 (H_3-20), 1.25 (H_3-22)]. The ^{13}C-NMR exhibits 24 carbon signals including signals assigned to two carbonyl carbons [δ_C 170.6 (C-19), δ_C 174.4 (C-23)], two olefinic carbons [δ_C 124.4 (C-11), δ_C 131.8 (C-12)], two oxygenated carbons [one methine (δ_C 83.0, C-3) and one methoxycarbonyl (δ_C 51.1, C_{19}-OMe)]. The signals originating from C-15 and C-17 was not observed owing to the fast equilibrium between the C-17-oxo-C-15-hydroxy, and C-17-hydroxy-C-15-oxo tautomeres. Analogue findings have been reported for other andrastins (*1*, *3*). The gross structure of **2**

was determined by detailed analyses of one and two dimensional NMR spectra. From COSY data we inferred the presence of three partial structures; X-CH_2-CH_2-CH-X; X-CH_2-CH_2-CH-O-X and -CH-CH= . The HMBC cross peaks revealed these fragments to form part of the skeleton of ring A, B and C. The $^{3}J_{H\text{-}C}$ established the presence of geminal methyl groups (C-24 and C-25) on the A ring together with a lactone bridge.

Supplementary Figure 1. Important HMBC connectivities in (**2**).

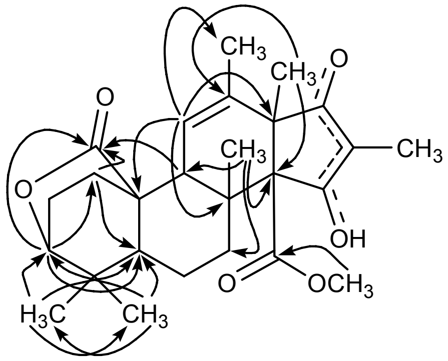

2

The low field shift of H-3 (δ 4.07) placed the oxygen on C-3. The HMBC connectivities between H-1b, H-3, H-5 and C-23 established the γ-lactone ring bridging C-3 and C-10. Broad carbon signals (C-14 and C-16) and the high field shifted carbon at δ 6.9 (C-18) are indicative of the presence of keto-enol tautomerism at the cyclopentane ring (ring D) as in andrastin A, B, C and D (*1*, *3*). The remaining elements of **2** were connected as shown in Figure 2 in accordance with the HMBC connectivities observed.
